# Wildlife trade investigations benefit from multivariate stable isotope analyses

**DOI:** 10.1111/brv.13175

**Published:** 2024-12-27

**Authors:** Tracey‐Leigh Prigge, Astrid A. Andersson, Chloe E. R. Hatten, Even Y. M. Leung, David M. Baker, Timothy C. Bonebrake, Caroline Dingle

**Affiliations:** ^1^ School of Biological Sciences, Kadoorie Biological Sciences Building The University of Hong Kong Pok Fu Lam Road Hong Kong SAR China; ^2^ Biology Department Capilano University 2055 Purcell Way North Vancouver British Columbia V7J 3H5 Canada

**Keywords:** stable isotope analysis, wildlife trade, wildlife forensics, captive–wild, geographic origin, species identification, validation

## Abstract

The investigation of wildlife trade and crime has benefitted from advances in technology and scientific development in a variety of fields. Stable isotope analysis (SIA) represents one rapidly developing approach that has considerable potential to contribute to wildlife trade investigation, especially in complementing other methods including morphological, genetic, and elemental approaches. Here, we review recent progress in the application of SIA in wildlife trade research to highlight strengths, shortcomings, and areas for development in the future. SIA has shown success in species identification, determination of geographic provenance, and differentiating between captive‐bred and wild individuals. There are also emerging applications of SIA in wildlife trade research including the use of labelling for traceability, more in‐depth analyses such as compound specific isotope analysis (CSIA), the use of trace metal isotopes, and monitoring the health of individuals (e.g. dietary history and nutritional status). While these applications have shown the utility of SIA in wildlife trade investigations, there are a number of limitations and issues where standardisation of analytical procedures would improve the comparability and interpretation of results. First, there is high variation within many stable isotopes geographically and within tissues – this variation presents opportunities for tracking and monitoring but can also challenge detection of patterns when variation is high. Second, the choice of isotopes and tissues within an organism (and ideally, multiple isotopes and tissues) should be considered carefully as different isotopes and tissue types have variable strengths and weaknesses depending on the research question. Third, validation of SIA methods remains underutilised in the field but is critical for applying SIA broadly to wildlife trade investigations and, particularly, for applications in forensics and in court. Fourth, standards are essential for comparisons across studies. Fifth, while some reference databases exist for the use of SIA in wildlife trade research (e.g. ivory), there are still few comprehensive reference databases available. Development of robust reference databases should be a priority for advancing the use of SIA in wildlife trade research, and ecological study more broadly. Ultimately, further recognition of these primary challenges (and development of solutions) within wildlife SIA research will improve the potential for this technique in tackling the threat of overexploitation to global biodiversity – particularly in concert with the application of other investigative techniques such as genetics and elemental analysis.

## I. INTRODUCTION

Global wildlife trade is a lucrative business, fuelled by the demand for wildlife as food, medicine, furniture, fashion, and pets (Andersson *et al*., 2021b). Legal wildlife trade has been estimated at averaging US $220 billion per annum between 1997 and 2017 (Andersson *et al*., 2021b). For illegal trade in wildlife, estimates are often cited in the literature, but vary greatly in magnitude, ranging from approximately US $5 to US $23 billion a year (e.g. Warchol, 2004; Alacs *et al*., 2010; Nellemann *et al*., 2014). While difficult to derive an exact value for wildlife trade (Sas‐Rolfes *et al*., 2019), it is clear that it is a multibillion dollar industry that has devastating impacts on wildlife populations, species and ecosystems (Scheffers *et al*., 2019; Morton *et al*., 2021; Mozer & Prost, 2023). In addition, trade in wildlife has been linked to the spread of zoonotic and agricultural diseases, leading to serious public health and economic concerns (Bezerra‐Santos *et al*., 2021; Cardoso *et al*., 2021).

Both local and international legislation exists to mitigate the negative impacts of wildlife trade. As an example, the Convention on International Trade in Endangered Species of Wild Fauna and Flora (CITES) is an international agreement between government signatories, regulating the global trade of threatened populations/species through a permit system (CITES, 2023). While CITES itself is not legally binding, it provides a framework for member parties to draft national laws in compliance with the treaty, prohibiting or restricting the import, export, and re‐export of CITES‐listed species (CITES, 2023). In addition to CITES, many countries/legislative regions have their own laws regulating the trade of wildlife (Ogden, Dawnay & McEwing, 2009). When investigating wildlife crimes, questions are often asked with relevance to legislation and may include (*i*) *identification of species* when only part of the genus is trade‐restricted, (*ii*) *identification of provenance* when only certain populations are trade‐restricted, and (*iii*) *determination of whether an individual is wild‐caught or captive‐bred/cultivated* as the trade of captive‐bred animals/cultivated plants, but not wild individuals, may be allowed for some species. To determine the legality of a sale, forensic evidence should be gathered and scientific methods should be used to address these questions.

A variety of different methods are widely used to address these questions related to wildlife trade investigations and wildlife forensics [e.g. stable isotope analysis (SIA), genetics, morphology, and elemental methods; Table 1; see online Supporting Information, Table S1, for cost estimates]. Morphology is commonly used for species identification, while genetics is used for both species identification and investigations into geographic provenance of traded individuals (Alacs *et al*., 2010; Ogden & Linacre, 2015; Trail, 2021). While these methods have provided important data, each has limitations. Morphological methods rely on the presence of distinguishing characteristics and genetics is reliant on DNA of sufficient quality. Samples encountered in wildlife trade investigations can be old and are often processed – DNA may be degraded/absent and distinguishing characteristics may have been removed. In such cases, alternative methods, used either on their own or in combination, can provide additional data and enable a greater understanding of the relevant issues. SIA is a relatively underused method, with potential for application in wildlife trade investigations and wildlife forensics. Since SIA does not rely on morphology or DNA, it can effectively address questions in cases where distinguishing characteristics have been lost or when DNA is degraded or absent in a sample (Ehleringer & Matheson, 2007).

**Table 1:** General comparison of stable isotope analysis (SIA) with other methods commonly used in wildlife trade investigations/wildlife forensics (Bowen *et al.*, 2005; Dormontt *et al.*, 2015; Ogden & Linacre, 2015; Nganvongpanit *et al.*, 2016; Hobson *et al.*, 2019a; Trail, 2021; McLellan *et al.*, 2022). N, no; Y, yes.

	SIA	Genetics	Morphology	Elemental methods, e.g. X‐ray fluorescence
Can be used if no DNA is present	Y	N	Y	Y
Can be used if DNA is degraded	Y	Sometimes	Y	Y
Can be used where there are no distinguishing morphological characteristics	Y	Y	N	Y
Captive/wild differentiation	Y	N	Sometimes	Y
Geographic provenance	Y	Y	N	Y
Genus identification	Y	Y	Y	Y
Species identification	Y	Y	Y	Y
Individual identification	N	Y	Y	N
Can be conducted *in situ*	Sometimes	N	Y	Y
Amount of sample required	As little as 1 ng	Approx. 100 mg	Must contain distinguishing features	Must cover device sample window (0.5 × 1.0 in)
Approximate price per sample (USD)^a^	$7–400	$8–300	<$100	~$130
Speed of process^b^	1–5 days	Several days	Minutes to a few days	10 s to a few minutes

^a^ Price per sample estimations were obtained using figures mentioned in the literature or quoted by laboratories offering these services in combination with the cost of services used by the author in 2024. The range used was compiled from the lowest and highest possible costs identified (see Table S1).

^b^ Estimate is for analysis/measurement only and does not include sample preparation.

SIA has been used widely in ecological studies to address a variety of questions related to animal diets/habitats and the origin of migrating animals (Hobson & Clark, 1992; Bearhop *et al*., 2003; Hebert *et al*., 2008; Bowen & West, 2019; Hobson *et al*., 2019a). While SIA has been used in criminal investigations, and has been applied in human forensics, it is still underused in wildlife forensic science (Ehleringer *et al*., 1999, 2000; Bowen, Wassenaar & Hobson, 2005; Meier‐Augenstein, 2010; Hinsley, King & Sinovas, 2016). Stable isotopes can be used to address questions related to wildlife trade and can provide additional data, expanding on the limitations of other methods (Chesson *et al*., 2018; Hinsley *et al*., 2016). More work is needed, however, to understand factors that impact stable isotope values in wildlife and to validate SIA for use in forensic case work.

In this review, we provide an overview of SIA, its use in wildlife trade research to date, and explore additional ways in which SIA can be applied in a wildlife trade context, drawing insights from other fields of research. Three main areas of research where SIA has potential for use in detecting wildlife crime include: (*i*) species identification; (*ii*) determination of geographic provenance; and (*iii*) differentiating between captive‐bred/cultivated and wild individuals. Furthermore, our review covers potential applications and limitations of SIA in wildlife trade research and wildlife forensics, with recommendations for future research needed to develop SIA further for use as a forensic tool.

### (1) Stable isotope analysis (SIA) background

Stable isotopes are non‐radioactive atoms of the same element that have different atomic masses (due to differing numbers of neutrons) and do not decay over time (Fry, 2006). Carbon (C), nitrogen (N), sulphur (S), oxygen (O) and hydrogen (H) are the most abundant elements in living organisms and together make up almost 100% of the dry mass of all plant and animal tissues (Wassenaar, 2019). These five elements are essential macronutrients and are considered ‘light elements’ as they are all relatively high in the periodic table and have low atomic mass. Nitrogen is an essential component of proteins, enzymes and nucleic acids. Carbon forms the backbone for many important molecules including proteins, nucleic acids and lipids and is essential for energy and storage. Oxygen and hydrogen are present in a variety of macromolecules and sulphur is present in just a few amino acids. Because of their abundance, as well as the availability of techniques to carry out mass spectrometry, the stable isotopes of these five macronutrients are widely used in wildlife trade investigations, offering valuable information on diet, habitat, and/or location (Hobson, 1999; Meier‐Augenstein, 2019; Table 2). Stable isotopes of these elements can be referred to as either light or traditional stable isotopes (we use traditional throughout).

**Table 2:** The five most commonly used stable isotopes (carbon, nitrogen, sulphur, oxygen and hydrogen), along with symbols for their values and ratios and sources of variation/signature change.

Stable isotope	Value/ratio	Sources of variation/signature change (Hobson, 1999; Fry, 2006; Wassenaar, 2019)	Ecology^a^		Wildlife trade investigations	
			Applications	Examples	Applications	Examples
**Carbon**	*δ* ^13^C (^13^C/^12^C)	– Photosynthetic pathways of primary producers (C3/C4) – Environmental conditions (e.g. water stress, CO_2_ source, light intensity)	– Assessing diet and habitat use – Migration studies – Food web studies	Birds (diet and habitat use): Bearhop *et al*. (2003) Dolphins (diet): Owen *et al*. (2011) Turtles (diet): Sung *et al*. (2021) Birds (migration): Chamberlain *et al*. (1996)	– Captive/wild – Provenance (if habitats differ) – Species identification	Cockatoos (captive/wild): Andersson *et al*. (2021a) Geckos (captive/wild): Dufour *et al*. (2022) Elephant ivory (provenance): van der Merwe *et al*. (1988); Ziegler *et al*., 2016 Cod (species identification): Oliviera *et al*. (2011) Scallops (species identification): Zhang *et al*. (2019)
**Nitrogen**	*δ* ^15^N (^15^N/^14^N)	– Trophic level – Associations with mycorrhizal fungi, bacteria in root nodules – Fertilisers in soil	– Assessing habitat and diet – Investigating trophic level	Birds (diet and habitat use): Bearhop *et al*. (2003) Dolphins (trophic level and diet): Owen *et al*. (2011) Bats (trophic level): Siemers *et al*. (2011) Turtles (diet): Sung *et al*. (2021)	– Captive/wild – Species identification	Cockatoos (captive/wild): Andersson *et al*. (2021a) Geckos (captive/wild): Dufour *et al*. (2022) Elephant ivory (provenance): Vogel *et al*. (1990) Elephant ivory and bone (provenance): Ziegler *et al*. (2016) Cod (species identification): Oliviera *et al*. (2011) Scallops (species identification): Zhang *et al*. (2019)
**Sulphur**	*δ* ^34^S (^34^S/^32^S)	– Biological processes (e.g. microbial activity in soil) – Atmospheric sources (e.g. sea spray, pollution, acid rain)	– Differentiating between individuals from marine, freshwater and terrestrial habitats	Fish: Fry & Chumchal (2011) Birds: Hebert *et al*. (2008)	– Cultivated/wild – Provenance	Cycads (cultivated/wild): Retief *et al*. (2014); Elephant ivory (provenance): Ziegler *et al*. (2016); Hale *et al*. (2021) Timber (provenance): Boeschoten *et al*. (2023)
**Oxygen**	*δ* ^18^O (^18^O/^16^O)	– Local environmental water sources – Latitude – Longitude – Altitude – Temperature – Distance to the open sea	– Migration studies – Geographic origin – Food web studies	Birds (migration): Hobson *et al*. (2004) Insects (migration): Hobson *et al*. (2012) General (food web): Vander Zanden *et al*. (2016)	– Provenance – Species ID	Birds (provenance): Hobson *et al*. (2004) Elephant ivory (provenance): Ziegler *et al*. (2016) Cedar trees (species identification): Paredes‐Villanueva *et al*. (2022) Timber (provenance): Boeschoten *et al*. (2023)
**Hydrogen**	*δ* ^2^H (^2^H/^1^H)	– Local environmental water sources – Latitude – Longitude – Altitude – Temperature – Distance to the open sea	– Migration studies – Geographic origin – Food web studies	Birds (migration): Chamberlain *et al*. (1996); Hobson *et al*. (2004) Insects (migration): Hobson *et al*. (2012) General (food web): Vander Zanden *et al*. (2016)	– Provenance	Birds (provenance): Hobson *et al*. (2004) Elephant ivory (provenance): Ziegler *et al*. (2016) Timber (provenance): Boeschoten *et al*. (2023)

^a^ Some current applications and examples of use in ecology for each of these isotopes are provided (this is not a comprehensive list but rather includes examples that relate to/can be applied to wildlife forensics).

Stable isotope values are expressed in *δ* notation and are not absolute abundance measurements. *δ* values measured during mass spectrometry are provisional as they are relative to international standards used during analysis. Measurements need to be normalised/calibrated to international standards before being reported (Meier‐Augenstein & Schimmelmann, 2019). The *δ* value is calculated as: [*δ* = (*R*_SAMPLE_/*R*_STANDARD_) − 1], where *R* is the ratio of the heavy isotope to the light isotope for the element. Delta (*δ*) values are expressed in parts per thousand (per mil), with the symbol ‰ (Fry, 2006; Szpak, Metcalfe & Macdonald, 2017).

In addition to analysis of traditional isotopes, many other elements also have isotopes that can be measured, including trace elements and heavy metals. Analysis of these non‐traditional elements has been used less commonly due to low abundance and additional challenges in extracting and analysing these isotopes. In addition, isotopes of trace elements/heavy metals can be easily contaminated and are usually more expensive to measure (Wassenaar, 2019). Strontium (Sr) and lead (Pb) are the most common examples of trace elements or metals that may be useful in wildlife trade investigations. Both of these elements are affected by variations in geological substrates and can show interesting geographic patterns (Hobson, 1999). Stable isotopes of trace metals are usually reported as ratios rather than as a *δ* value.

Within individual organisms, geographic/habitat variation is the main cause of differences in stable isotope composition, as stable isotopes are acquired through the consumption and assimilation of food and water from the surrounding environment (Hobson, 1999). Isotope ratios of each element can be impacted by multiple processes (Hobson & Wassenaar, 2019). *δ* ^13^C values vary due to differences in the photosynthetic pathways of plants (C3 *versus* C4) at the bottom of the food web, while *δ* ^15^N values vary depending on the trophic level of the species being measured. *δ* ^15^N values are also impacted by different types of pollution, including sewage or agricultural runoff. *δ* ^34^S values are influenced by the amount of sulphur in the local environment (Hobson, 1999; Fry, 2006). *δ* ^2^H values are usually less negative in coastal/equatorial regions and more negative in inland, high‐altitude or high‐latitude regions (Hobson, 1999; Fry, 2006; Meier‐Augenstein, 2019). *δ* ^18^O values in water and plants vary with temperature and amount of precipitation in the region (Hobson, 1999; Fry, 2006). Water is taken up by plants or consumed by animals and will have a different isotopic composition based on location. *δ* ^2^H/*δ* ^18^O values of plants should be closely correlated with their water source, while *δ* ^2^H/*δ* ^18^O values of animals will reflect both drinking water and water obtained from their food.

Within an individual, stable isotope values can vary among tissues due to differences in turnover rates. Living tissues are in a continuous state of degradation and synthesis, resulting in renewal over time (tissue turnover). Different tissues have different turnover rates (the length of time taken for the tissue to be completely renewed), which affects the timeframe over which environmental/dietary information is captured and incorporated (Hobson, 1999). Tissues with rapid turnover rates (e.g. blood plasma and liver) can provide information on recent events [days to weeks (Hobson & Clark, 1992; Evans Ogden, Hobson & Lank, 2004; Sponheimer *et al*., 2006)] while tissues with slower turnover rates (e.g. red blood cells, muscle, skin, teeth and bone) reflect longer time periods [months to years (Hobson & Clark, 1992; Sponheimer *et al*., 2006)]. Some tissues are biochemically inert after synthesis (e.g. keratin, feathers) and the stable isotope composition at the time of synthesis may be fixed within them. This means that the stable isotope values will reflect the diet of the animal during tissue growth and remain stored until the tissue is replaced (Hobson, 1999; Bowen *et al*., 2005). Tissues that grow continuously with time‐dependent growth (e.g. hair, scales, and claws) can record incremental change over several years (Bearhop *et al*., 2003). Different sections of these tissues will represent a chronological record of the diet/health of the animal (Bearhop *et al*., 2003). It is possible to investigate changes over time in plants by comparing stable isotope values of older tissues to those that have grown more recently. For example, in cycads, the petiole and upper leaf base have the youngest plant tissue, with plant tissue age increasing towards the lower leaf bases (Retief, West & Pfab, 2014) and for timber with distinct tree rings, isotope values of the different tree rings can represent a time series reflecting different time periods in the tree's history (Kagawa & Leavitt, 2010).

Stable isotope values in tissues within the same organism can also vary due to differences in the routing of elements (and thus isotopic signals) during tissue synthesis. Different tissues incorporate elements from different sources in the diet (i.e. protein, carbohydrate or lipid) which can have a significant effect on their stable isotope values. Bone collagen, for example, primarily incorporates the protein component from an animal's diet while the structural component of bone (bioapatite) incorporates elements from the whole diet (DeNiro & Epstein, 1978; DeNiro & Epstein, 1981b). Because of this, stable isotope values measured in collagen might differ from those measured in bioapatite. Heavy and light isotopes of an element are incorporated into tissues at different rates due to differences in mass and bond strength (isotopic fractionation), leading to differences in isotopic ratios between food sources and tissue. In most cases, the light isotopes will be preferentially incorporated into tissues, but in some chemical reactions the heavy isotopes may instead be favoured (Fry, 2006). In addition to leading to differences in stable isotope values within an individual, this effect of isotopic fractionation increases across higher trophic levels (DeNiro & Epstein, 1978; Minagawa & Wada, 1984; Vander Zanden & Rasmussen, 2001).

### (2) Stable isotope analysis (SIA) workflow

**Fig. 1:**
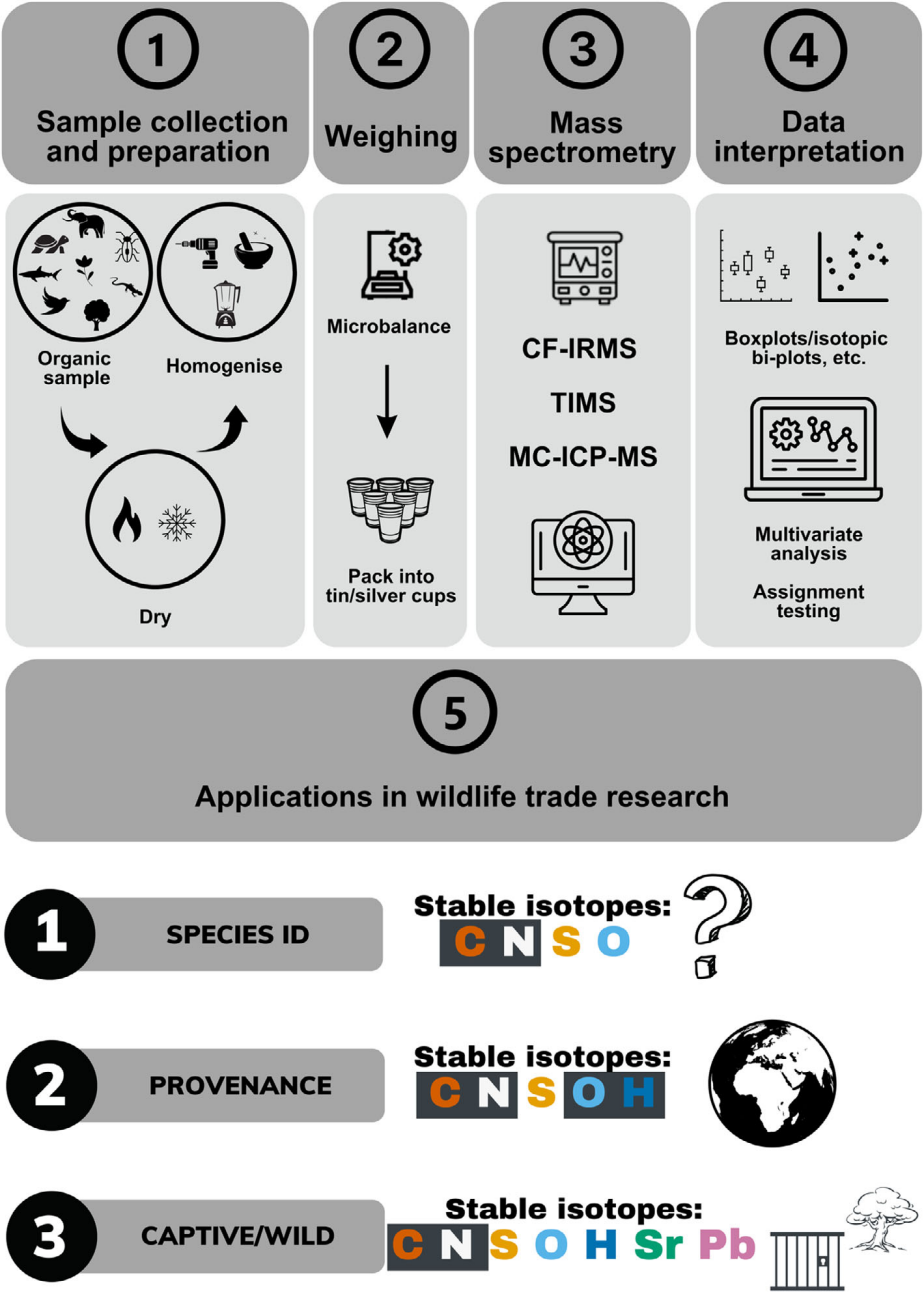
General workflow for bulk stable isotope analysis (SIA). (1) Sample collection and preparation: sampling, drying (in an oven or freeze drier), and homogenisation (with a mortar and pestle, drill, or blender). (2) Weighing, followed by packing into tin/silver cups. (3) Mass spectrometry using, for example, a continuous flow isotope ratio mass spectrometer (CF‐IRMS), thermal ionization mass spectrometer (TIMS) or multicollector inductively coupled plasma mass spectrometer (MC‐ICP‐MS). (4) Data interpretation, e.g. data plotting and statistical analysis. (5) Application of SIA to wildlife trade research in three main areas (species ID, provenance and captive/wild). Stable isotopes that have been used most commonly for each area are shaded, stable isotopes that have been used to a lesser extent are not shaded (C: carbon; N: nitrogen; S: sulphur; O: oxygen; H: hydrogen; Sr: strontium; Pb: lead). Note that steps and methods for analysis may vary according to individual protocols and aims.

The SIA workflow generally consists of five main steps (sample collection and preparation, weighing, mass spectrometry, data interpretation, and applications; Fig. 1). Depending on the samples used and purposes of the investigation, additional steps may be required. Preparation of samples for SIA processing is generally straightforward and often does not require any additional specialised equipment. However, specialised equipment may be required in some cases, e.g. a Soxhlet extractor for defatting or degreasing samples or a centrifuge for extracting lipids or collagen. At present, there are a number of laboratories worldwide offering a service where samples can be submitted for SIA processing for a fee e.g. The Stable Isotope Ratio Mass Spectrometry Laboratory at The University of Hong Kong, the UC Davis Stable Isotope Facility, and The Stable Isotope Laboratory at Washington State University.

### (3) Applications of stable isotope analysis (SIA)

Ecological research has benefitted from the use of SIA techniques in various ways, including investigations of trophic level, migration/geographic origin, habitat use, and dietary composition (Table 2; Hobson, 1999; Fry, 2006; McKechnie 2006; Meier‐Augenstein, 2019). The same techniques that have helped ecologists investigate diet can be applied forensically to determine whether individuals have captive or wild origins, and those used for understanding migration patterns may help forensic scientists identify the geographic origins of trafficked individuals/products (Chesson *et al*., 2018).

SIA has been used to investigate crime and has provided forensic evidence used to prosecute crimes (Matos & Jackson, 2019). SIA is an official method accepted by the Association of the Official Agricultural Chemists (AOAC) International for food forensics, used for investigating the authenticity of food and flavours, identifying contamination, and tracing provenance (Danezis *et al*., 2016; Camin *et al*., 2017; Chesson *et al*., 2018). SIA has also been used in multiple criminal investigations, helping to identify both victims and perpetrators, as well as origins of narcotics, explosives, and counterfeit currency (e.g. Ehleringer *et al*., 1999, 2000; Ehleringer & Matheson, 2007; Meier‐Augenstein & Fraser, 2008; Lehn, Rossmann & Graw, 2015; Cerling *et al*., 2016; Danezis *et al*., 2016; Camin *et al*., 2017; Chesson *et al*., 2018; Meier‐Augenstein, 2019). To develop further the use of SIA in forensics, Ehleringer & Matheson (2007) published a guide for lawyers and courts, including potential forensic applications and limitations. SIA methods have been validated for forensic use in criminal cases, e.g. Mekota *et al*. (2006) developed a method for the use of serial analysis of *δ* ^13^C and *δ* ^15^N values in hair samples to identify starvation in humans. This method was validated for forensic use in a follow‐up study and can now be used to provide evidence in cases where malnutrition/starvation has been caused by abuse/neglect (Mekota *et al*., 2009).

SIA has been applied to wildlife forensics only sporadically; it is not commonly practiced and its use has not been well documented. Three main areas where SIA has been applied in wildlife trade investigations include species identification, determination of geographic provenance, and differentiation between captive‐bred/cultivated and wild individuals (Fig. 1).

## II. SPECIES IDENTIFICATION

When investigating wildlife crimes, it is important to identify species to ensure the application of appropriate enforcement measures. Taxa can be identified to species level using morphology (e.g. macromorphology, microscopic analysis, Schreger pattern analysis, wood anatomical analysis), genetics (e.g. barcoding, population genetics/phylogeography), and/or elemental methods (e.g. mass spectrometry, near‐infrared spectroscopy) (Ogden *et al*., 2009; Dormontt *et al*., 2015; Baker *et al*., 2020; Trail, 2021). Each of these methods, however, has limitations. For example, identification by morphology is not always possible with processed samples. Once processed, timber no longer possesses the components (e.g. leaves, flowers, fruit) used for plant identification (Dormontt *et al*., 2015). Similarly, wildlife derivatives that have been made into jewellery, fashion items, or medicine have often lost identifying characteristics. Identification *via* genetic methods also can be difficult/impossible with processed or degraded samples. These kinds of samples are often encountered in the field of wildlife forensics and an alternative or complementary method may be helpful.

Although SIA methods are not commonly leveraged for species identification – especially in wildlife forensics – there is potential that methods can be developed for some species, or at least to exclude certain species where necessary. To maximise effectiveness, SIA for species identification/exclusion should be used in scenarios where the species of interest have different distributions and/or diets. In addition to variation caused by differences in range and habitat/diet, there may also be species‐specific differences in isotope values not related to location, habitat, or diet which could be exploited for species identification. For example, differences were observed in stable isotope values in Burmese (*Python bivittatus*) *versus* reticulated pythons (*Python reticulatus*) even though their distributions and diets overlapped. A possible explanation for this variation could be differences in body size, which results in individuals of these two species occupying different trophic niches within the habitats they occupy (Natusch *et al*., 2017).

SIA has been used for the identification of seabirds, timber trees, and seafood (Table S2). In animals, *δ* ^13^C and *δ* ^15^N values have been used most widely for this purpose, as values have been found to differ between species, even when the origin was the same (Oliviera *et al*., 2011; Militão *et al*., 2014; Zhang *et al*., 2019). In plants, *δ* ^13^C and *δ* ^18^O values have been used successfully to differentiate between two species of *Cedrela* (*C. odorata* Linn. and *C. fissilis* Vell.) (Paredes‐Villanueva *et al*., 2022). SIA can also potentially be used to differentiate between species to detect laundering. In most countries, woolly mammoth (*Mammuthus primigenius* Blumenbach) ivory is legal to trade while elephant (*Loxodonta africana* Blumenbach/*L. cyclotis* Matschie or *Elephas maximus* Linn.) ivory is not. The ranges and diets of elephants and woolly mammoths differ greatly, so SIA can likely be used to differentiate between these two types of ivory.

## III. DETERMINATION OF GEOGRAPHIC PROVENANCE

Knowledge of the geographic origin of an organism/wildlife product is important in wildlife crime investigations. From a legal perspective, for trafficked species with a broad geographic range encompassing multiple countries, it is important to determine the provenance of seized samples so that the correct legislation can be used when prosecuting these crimes (Ogden & Linacre, 2015). From an enforcement perspective, information on provenance can help to highlight poaching hotspots or trafficking routes for law enforcement so that trade pathways can be effectively targeted and disrupted. However, there are limitations to the applicability of current methods for determining provenance (Hobson, 2003; Hobson *et al*., 2004; Ogden & Linacre, 2015). Genetic methods require DNA of sufficient quality, while data generated by studies involving tagging/marking are often geographically biased – with rate of recovery proportional to sampling/tagging effort at specific locations (Wassenaar & Hobson, 2001; Hobson, 2003). Remote‐sensing techniques are often expensive and limited to larger bodied and/or high profile species (Hobson, 2003).

Stable isotopes provide a supplementary method that can add data informative for identifying the geographic provenance of an individual/product. Hydrogen and oxygen stable isotope values (*δ* ^2^H and *δ* ^18^O) show considerable, relatively predictable geographic variation which can be used to construct global maps of isotopic distributions across landscapes (isoscapes). Isoscapes have been developed for *δ* ^2^H and *δ* ^18^O values using environmental samples, e.g. precipitation, leaf water, and vegetation (www.waterisotopes.org; Bowen & Wilkinson, 2002; West, Sobek & Ehleringer, 2008; West *et al*., 2010; Bowen *et al*., 2014). Data from isoscapes can be interpolated to enable comparison to stable isotope values from plant/animal tissues to investigate provenance (Vander Zanden *et al*., 2018). This has been carried out using interpolation from precipitation to chemically inert animal tissues such as bird feathers (Bowen *et al*., 2005) and dragonfly wings (Hobson *et al*., 2012). Isoscapes provide accurate estimates for *δ* ^2^H and *δ* ^18^O values that can be compared to animal tissues of known/unknown origin. They could potentially be applied to a wider range of biological materials/tissue types (i.e. samples from other species), however, this would require calibration with samples of known origin for each new tissue type.

Once an isoscape has been developed successfully, a tool can be developed to map different seized products to their locations of origin. Online mapping tools using SIA data have been developed for elephant ivory (www.ivoryid.org) and are currently being developed for timber in combination with other methods, e.g. DNA fingerprinting (www.worldforestid.org). However, the amount of data available varies both temporally and spatially, limiting the use of these maps/tools. As an example, IvoryID currently contains data from 694 African elephant (*L. africana*) samples representing 29 African countries. However, there are fewer than five geographic reference samples of known origin for 13 of these countries and, therefore, isotopic profiles from these regions may not be very precise (Ziegler, 2017). For investigating a sample with a specific isotopic fingerprint, a lack of similar isotopic fingerprints in the database leads to limited confidence in the findings and they would not be sufficiently robust for use in a criminal investigation (Hale *et al*., 2021).

In animals, SIA has been used to investigate geographic provenance for multiple traded taxa over the past 35 years (Table S3). Most studies to date have focused on elephant ivory (e.g. van der Merwe, Lee‐Thorp & Bell, 1988; Vogel, Eglington & Auret, 1990; Cerling, Omondi & Macharia, 2007; Ziegler *et al*., 2016; Hale *et al*., 2021), but there have also been some involving different bird species using feathers (e.g. Hobson *et al*., 2004; Kelly, Thompson & Newton, 2008; Haché *et al*., 2012) and, more recently, turtles, Philippine pangolin (*Manis culionensis*), and cheetah (*Acinonyx jubatus*) using scutes, claws, scales, and hair (Brandis *et al*., 2023; Koehler *et al*., 2023). Overall, carbon stable isotopes are useful for differentiating between different populations when differences in habitat exist [e.g. forested *versus* grassy (Ambrose & DeNiro, 1986; van der Merwe *et al*., 1988; Vogel *et al*., 1990; Cerling *et al*., 2007; Ziegler *et al*., 2016; Hale *et al*., 2021; Brandis *et al*., 2023)] and oxygen and hydrogen stable isotopes have been useful when there are differences in water sources used by different populations (Hobson *et al*., 2004; Cerling *et al*., 2007; Kelly *et al*., 2008; Haché *et al*., 2012; Ziegler *et al*., 2016). In some captive/wild investigations, the inclusion of hydrogen and/or oxygen stable isotopes has facilitated the discernment of geographic provenance in addition to differentiating between captive and wild individuals (e.g. Dittrich, Struck & Rödel, 2017; Natusch *et al*., 2017; Alexander *et al*., 2019; Jiguet, Kardynal & Hobson, 2019). Some animals (e.g. elephants), however, are ecological generalists and feed on a wide variety of vegetation types, and stable isotope values will be correspondingly diverse. Leveraging a multi‐isotope approach can help improve the precision of origin estimates in such cases. Additional isotopes that have proved useful for such investigations are nitrogen, sulphur, strontium and lead stable isotopes (Ambrose & DeNiro, 1986; Vogel *et al*., 1990; Ziegler *et al*., 2016; Hale *et al*., 2021; Brandis *et al*., 2023).

In plants, SIA has been used to investigate geographic provenance for a number of species (Table S3), but with varied results. *δ* ^13^C values have been useful for separating wood from different geographic regions in some cases (Kagawa & Leavitt, 2010), but have shown overlapping values for some different regions (Austria and Siberia) where provenance could not be determined using carbon stable isotopes alone (Horacek, Jakusch & Krehan, 2009). *δ* ^2^H and *δ* ^18^O values have also been used successfully as they are influenced by the water sources of the plant, which vary between different geographic regions (Keppler *et al*., 2007; Horacek *et al*., 2009). Element concentrations of sulphur and lead vary according to pollution levels and can potentially be used to differentiate between plants growing in polluted *versus* non polluted locations (Trust & Fry, 1992; Komárek *et al*., 2008; Zheng *et al*., 2023). Isoscapes of carbon, sulphur, oxygen and hydrogen stable isotopes have been developed for oak trees in the USA to determine origin (Watkinson *et al*., 2020). Due to high local variability, some more recent studies in central Africa and South America have demonstrated that SIA is useful for country‐level assignment of timber but not for sub‐country origin assignment, even when using multiple isotopes (Vlam *et al*., 2018; Paredes‐Villanueva *et al*., 2022; Boeschoten *et al*., 2023). Therefore, it is currently not recommended to rely solely on SIA to determine the provenance of timber.

## IV. DIFFERENTIATING BETWEEN CAPTIVE‐BRED/CULTIVATED AND WILD INDIVIDUALS

The legality of trade often depends on whether an individual was captive‐bred/cultivated or wild‐sourced. However, it is usually impossible to distinguish between a captive‐bred or wild‐caught individual by visual examination, and current traceability methods can be unreliable. For example, government‐issued permits and trader declarations can be falsified, and only function well if there is sufficient enforcement (Rosen & Smith, 2010). For birds, closed metal leg bands with identifying information can be applied to captive‐bred chicks, but it is easy for poachers to apply these falsely to wild‐caught chicks brought into captivity (Ortiz‐von Halle, 2018). Similarly, microchips are sometimes used to mark wild plants, but poachers use scanners to detect and remove them (Retief *et al*., 2014). When differentiating between captive‐bred/cultivated and wild‐sourced individuals, SIA is a good alternative method as there is no tag/chip that can be removed or tampered with. SIA can also yield results of high quality even many years after the poaching event (Hinsley *et al*., 2016).

The premise for using SIA to differentiate captive‐bred/cultivated and wild‐sourced individuals is that differences in the diets and/or water sources of captive *versus* wild individuals result in differences in their stable isotope values. Animals born and raised in captivity are generally fed a fairly uniform diet of a few distinct taxa while diets of wild animals are more likely to contain a variety of taxa and to change according to the animals' physiology and environment. Because of this, wild animals tend to have a wider isotopic niche than captive animals (Hobson, 1999; Fry, 2006; van Schingen‐Khan *et al*., 2016; Brasileiro *et al*., 2023). Moreover, diets of captive‐bred animals often consist of commercial feeds containing agricultural products like corn (which is a C4 plant and therefore would have a *δ* ^13^C value between −12 and − 16 ‰) or wheat (which is a C3 plant and therefore would have a *δ* ^13^C value between −25 and − 29 ‰) (O'Leary, 1988). For example, in yellow‐crested cockatoos (*Cacatua sulphurea* Gmelin) in Hong Kong, captive‐bred birds had higher *δ* ^13^C values in comparison to wild birds, reflecting their diet of commercial parrot pellets which usually contain a high proportion of corn (Andersson *et al*., 2021a).

**Fig. 2:**
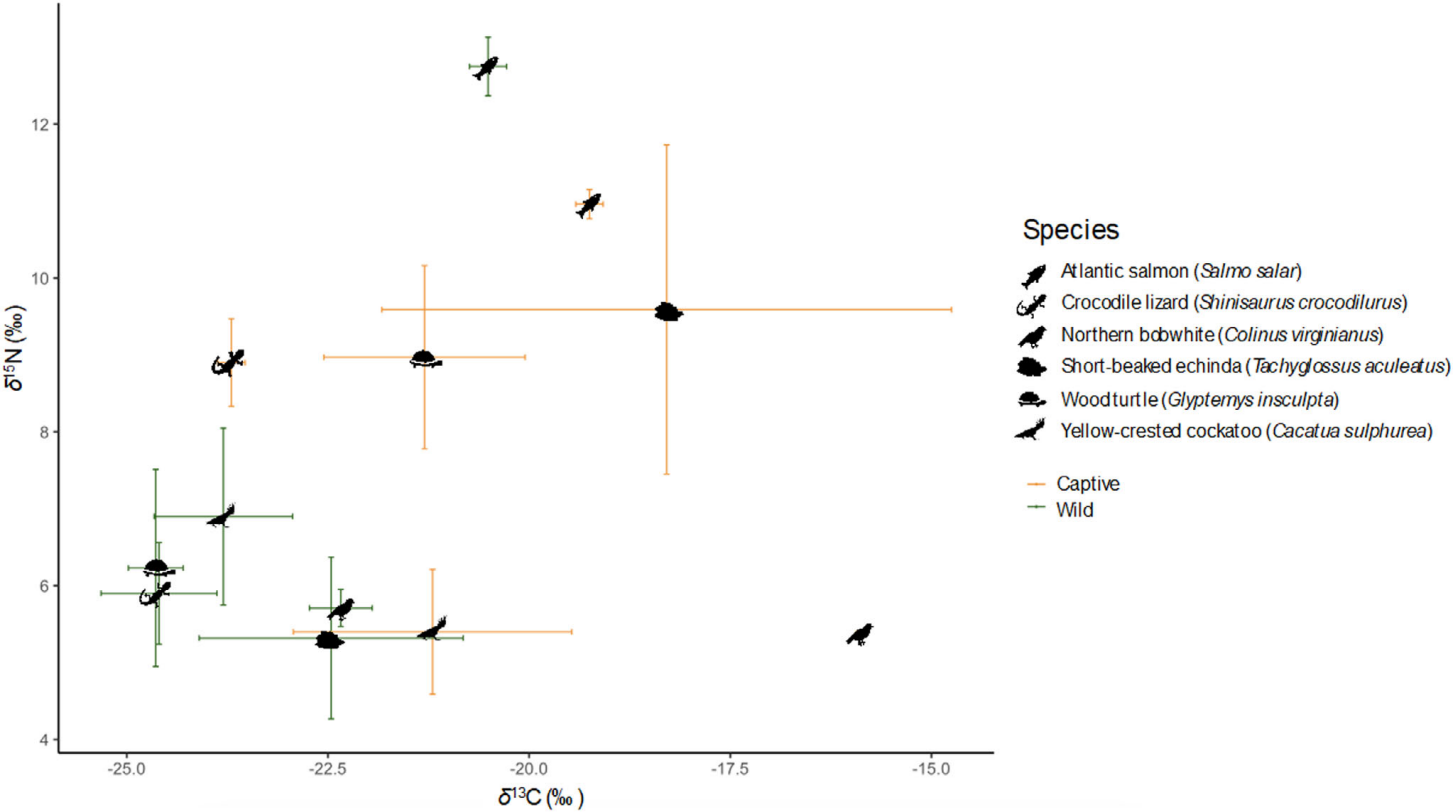
Ranges of *δ* ^13^C and *δ* ^15^N values for captive‐bred and wild‐caught animals based on previous studies (Dempson & Power, 2004; van Schingen‐Khan *et al*., 2016; Castelli & Reed, 2017; Brandis *et al*., 2018; Andersson *et al*., 2021a; Hopkins *et al*., 2022). Bars represent the ranges of the *δ* ^13^C and *δ* ^15^N values in each study. The *δ* ^13^C and *δ* ^15^N value ranges for the captive northern bobwhite samples were narrow and lie within the icon.

In the past 20 years, studies on multiple taxa have demonstrated differences in stable isotopes that effectively separate captive‐bred and wild‐caught individuals (Table S4). *δ* ^13^C and *δ* ^15^N values are most commonly used in these studies as they provide a direct measure of diet (e.g. Dempson & Power, 2004; van Schingen‐Khan *et al*., 2016; Castelli & Reed, 2017; Brandis *et al*., 2018; Andersson *et al*., 2021a; Hopkins *et al*., 2022). It is difficult to reproduce the natural diet of an animal in captivity, as captive and wild diets often differ greatly, and thus their *δ* ^13^C and *δ* ^15^N values can be used effectively to differentiate between captive‐bred and wild‐caught individuals (e.g. Dempson & Power, 2004; van Schingen‐Khan *et al*., 2016; Castelli & Reed, 2017; Brandis *et al*., 2018; Andersson *et al*., 2021a; Hopkins *et al*., 2022; Fig. 2). Hydrogen SIA has also been demonstrated to differentiate successfully between captive and wild individuals, reflecting differences in water sources of the two groups (Natusch *et al*., 2017; Alexander *et al*., 2019; Jiguet *et al*., 2019). One study included *δ* ^34^S in their analyses but, in this case, discrimination of captive *versus* wild individuals was found to be influenced mostly by *δ* ^13^C and *δ* ^15^N (Castelli & Reed, 2017).

In addition to differentiating between captive and wild animals, SIA has been used to differentiate between cultivated and wild plants (Table S4). Strontium isotope ratios are useful for this as they relate to local geology and very little fractionation occurs, therefore isotope ratios are fairly consistent across trophic levels (West *et al*., 2009b; Flockhart *et al*., 2015). Nitrogen varies by type of inputs, including different fertilisers used in soils. Because of this, *δ* ^15^N values are useful for differentiating between plants grown in unfertilised soils in the wild and plants grown in fertilised soils in cultivated gardens (West *et al*., 2009a). In a study investigating translocated cycads, a range of different tissues (newer post‐relocation tissues *versus* older pre‐relocation tissues) were analysed in two species to investigate whether plants originated from the wild before being moved to cultivated gardens (Retief *et al*., 2014). *δ* ^34^S values, *δ* ^18^O values and ^87^Sr/^86^Sr differed between relocated and control (wild) plants for Lebombo cycad (*Encephalartos lebomboensis* I. Verd), while differences were found in ^87^Sr/^86^Sr and *δ* ^15^N values between tissues grown pre‐relocation (which were the same as wild plants) and those grown post‐relocation for Alexandria cycad (*Encephalartos arenarius* R. A. Dyer). The results confirmed that both plants had been moved to a new location and indicated that strontium SIA, especially, is a powerful tool for differentiating between wild and cultivated plants (Retief *et al*., 2014).

## V. EMERGING DEVELOPMENTS IN APPLICATION

In addition to the applications mentioned above, SIA can be used in innovative ways to aid wildlife trade investigations. Potential uses include labelling individuals/products with isotopic markers to enable tracing, use of more in‐depth analyses such as compound specific isotope analysis (CSIA), use of trace metal isotopes, and using stable isotopes to investigate the health of individuals along trafficking pathways.

### (1) Labelling for traceability

There are ways in which SIA can be applied to label individuals of interest for improved traceability. One way to do this is by manipulating diets of captive‐bred individuals, thereby creating signatures that can be compared to reference databases when necessary. In this case, reference databases would consist of isotopic data for all possible feeds used for these captive‐bred individuals. As an example, controlled feeding experiments were carried out for snakes using five different diet treatments of foods commonly fed to snakes at farms in Asia (Natusch *et al*., 2017). Captive snakes that were fed a diet consisting predominantly of pork were never misclassified as wild (Natusch *et al*., 2017), indicating that, by feeding specialised diets to captive‐bred snakes, the stable isotope compositions can be manipulated to ‘label’ captive‐bred individuals. This could be further supported by the development of a reference database for breeding farms to enable comparison of any specimens where uncertainty of captive *versus* wild origin exists. Few studies have been conducted on this topic and future work should include these kinds of diet studies across different taxa.

Another option is to utilise isotopes in a proactive way by actively deploying them for tracing purposes. Specific isotopic tracers can be used in food/water to actively label individuals. This has been tested, for example, by using an ^15^N tracer (glycine) to label captive‐bred lizards isotopically (Crook, Musing & Ziegler, 2016). In this case, controlled feeding experiments were carried out in which lizards were fed mice that had been injected with the ^15^N marker and the time lag for the isotopic signal to appear in faeces and shed epidermis was examined. The label was detected in both shed skin and faeces within 2–3 weeks and was still present in shed skin more than 3 months after it was ingested. Similarly, different methods have been used to label plants isotopically, e.g. cultivation in labelling chambers, pulse‐labelling, root feeding, stem infiltration, and leaf tip feeding (Berg *et al*., 1991; Hertenberger & Wanek, 2004; Subke *et al*., 2009). These methods could potentially be applied in a wildlife trade context to label plants for simpler identification later on. To develop further the application of isotopic tracers in wildlife trade research, studies should focus on assessing the length of time it takes for the tracer to be incorporated into various tissues in different species. Furthermore, studies could be enhanced by monitoring how long a ‘wild’ signature remains within a tissue once the individual has been taken from a wild habitat and placed into captivity/cultivation.

### (2) Compound specific isotope analysis (CSIA)

When interpreting SIA results, one of the biggest challenges is determining whether the variation in results is due to changes in diet/trophic position, differences in fractionation, changes in the baseline isotope values, or a combination of these factors (Post, 2002). SIA typically measures the composition of an entire tissue sample (bulk SIA), providing a single value per isotope measured. CSIA, by contrast, measures the stable isotope values of individual amino acids (AAs) that make up the proteins within a tissue sample, providing a series of values for each sample, analogous to an ‘isotopic fingerprint’. One of the key benefits of CSIA‐AA is that a single sample can be used to measure both the baseline isotope values from the base of the food web and to obtain information on trophic position (McMahon & Newsome, 2019; Whiteman *et al*., 2019). Stable isotope values of AAs vary depending on the different biochemical pathways by which they are assembled. Essential AAs derive their carbon from proteins acquired through an animal's diet. As the carbon is assimilated, isotopic fractionation occurs, leading to a broad range of stable isotope values. Non‐essential AAs can be synthesised *de novo* by an animal and thus show a more uniform range of stable isotope values (Matos & Jackson, 2019). Comparing the isotopic composition of essential and non‐essential AAs can therefore provide information on an animal's diet compared to an inbuilt baseline (provided by the non‐essential AAs).

As both sample preparation and analysis are more complicated (and more expensive) than for bulk SIA, and different instrumentation is required, CSIA has not yet been used as widely as bulk SIA in ecology or in wildlife trade investigations. CSIA has been used more often in marine ecosystems, and there are relatively few studies using CSIA in terrestrial ecosystems, particularly in vertebrates. While some work has been done to investigate carbon and nitrogen stable isotope fractionation between the diet and the consumer in individual AAs (McMahon *et al*., 2015), more such studies are needed in order to build robust models for interpreting CSIA data. There are, however, a range of relevant questions that can be answered using this approach, including studies of movement between different ecosystems and a more detailed understanding of the source of an animal's diet (see references in McMahon & Newsome, 2019). CSIA has been used to increase the accuracy in distinguishing between captive and wild cockatoos in Hong Kong (Andersson *et al*., 2021a). In this case, *δ* ^13^C values differed significantly for six AAs between captive and wild cockatoos, enabling successful differentiation for samples that were ambiguous when using SIA alone (Andersson *et al*., 2021a). There is much potential for this technique in relation to wildlife crimes.

### (3) The stable isotope analysis of trace metals

Variation in stable isotopes of trace metals is influenced by different processes to those affecting traditional stable isotopes, therefore they can serve as complementary markers and their use can increase the spatial resolution of current estimates. Stable isotopes of trace metals occur naturally in smaller amounts than traditional isotopes and have been less commonly applied in biological research due to the expense of the required instrumentation and more complicated and time‐consuming sample preparation methods. In addition, traditional thermal ionization mass spectrometer (TIMS) techniques are slower and less efficient in processing samples. However, recent advances in the development of multiple collector inductively coupled plasma mass spectrometer (MC‐ICP‐MS) instrumentation make it possible to measure multiple isotopes simultaneously and with greater speed (Aggarwal, Habicht‐Mauche & Juarez, 2008; Bai *et al*., 2021). Strontium (Sr) and lead (Pb) stable isotopes provide independent geographic information with patterns corresponding to those of the local rocks and soils (Aggarwal *et al*., 2008). ^87^Sr/^86^Sr varies with differences in bedrock and different regions have distinct strontium stable isotope compositions which are dependent on the composition and nature of the rocks (Coelho *et al*., 2017). Lead isotopes closely reflect tectonic regimes and regional variation occurs due to variation in lead ores used in different regions (Evans *et al*., 2022). This regional variation could help distinguish location within a range of similar values obtained using traditional stable isotopes.

A major benefit when using strontium isotopes is that ratios generally remain constant across food webs. Strontium is easily absorbed and is not usually influenced by fractionation and/or external factors such as atmospheric sources, fertilisers or other anthropogenic inputs (West *et al*., 2009b; Coelho *et al*., 2017; Flockhart *et al*., 2015; Crowley, Miller & Bataille, 2017). This means that plants, and the animals feeding on them, often carry the same strontium isotopic composition as the local geology and this ratio can be used to track an animal's origin (Aggarwal *et al*., 2008). As ratios are consistent amongst different samples from the same region, ^87^Sr/^86^Sr of soil could be used to infer ratios for plants and animals inhabiting the same local area. A baseline strontium isoscape constructed from ^87^Sr/^86^Sr of soil samples would be beneficial because this could then be extrapolated to a variety of different plant and animal tissues. Care must be taken, however, as fractionation has been observed in some cases (de Souza *et al*., 2010). In this case, fractionation was caused by the plants (*Rhododendron* and *Vaccinium*) preferentially assimilating the lighter isotope of strontium (^86^Sr), resulting in lower ^87^Sr/^86^Sr ratios (de Souza *et al*., 2010). General trends, however, have shown no fractionation from soil across trophic levels in plants (Song *et al*., 2014), fish (Pouilly *et al*., 2014) and butterflies (Flockhart *et al*., 2015). When planning a new study, it is important to compare vegetation and herbivore ^87^Sr/^86^Sr with ratios of the soil/bedrock. If the ratio is consistent among samples, time and effort can be saved as intensive sampling efforts for the species of interest can be replaced/supplemented with finer scale sampling of plants and soils. Unlike strontium, primary lead signatures are affected by anthropogenic activities (e.g. industrial activities and leaded petrol usage) and this must be taken into consideration when planning a study as ratios will be affected (Kamenov & Gulson, 2014).

Stable isotopes of trace metals have not yet been widely applied in wildlife trade research, however, variations in ^87^Sr/^86^Sr have been used successfully to investigate origin and/or to track migration, e.g. for cycads (Retief *et al*., 2014), timber (English *et al*., 2001), fish (Pouilly *et al*., 2014), birds (Chamberlain *et al*., 1996), elephants (Vogel *et al*., 1990; Koch *et al*., 1995), and even extinct mammoths and mastadons (Hoppe *et al*., 1999). In timber, strontium ratios were used to narrow down origins to specific mountains in north‐western New Mexico due to differences in ^87^Sr/^86^Sr, in this case possibly due to the influence of atmospheric dust sources of strontium (English *et al*., 2001). Inclusion of strontium SIA could, therefore, aid in identifying timber origins where, until now, it has been difficult to move beyond country‐level origin assignment when using only traditional stable isotopes. Lead stable isotopes have been used to investigate habitat/origin for elephants (Vogel *et al*., 1990) and a lead isoscape has been created for Britain (Evans *et al*., 2022). As there is a major tectonic boundary separating Scotland from England and Wales, this isoscape was used successfully to narrow down provenance for Neolithic pigs from sites in southern England, where the use of strontium SIA alone had been unsuccessful. This study highlights the fact that strontium and lead can provide complementary information, and demonstrates the potential of using lead SIA for investigating provenance in cases where results may be ambiguous when using only strontium. It would be beneficial to develop this isoscape further to include other regions worldwide to facilitate more investigations in cases where results are ambiguous when using strontium and/or the traditional stable isotopes. In addition, exploration of other uses of trace metal stable isotopes in research that is not related to wildlife trade can now be used to inspire novel ways of using these methods in a different context.

### (4) Health

SIA can be used to detect nutritional stress (i.e. reduced food intake or low‐quality diet), dietary changes, and even disease (e.g. Hobson, Alisauskas & Clark, 1993; Mekota *et al*., 2006; Petzke *et al*., 2006; Huelsemann *et al*., 2009). A number of studies have investigated the influence of different nutritional stressors/changes and health conditions on isotope values of different animal tissues over the past 30 years (Table S5). For example, starvation increases *δ* ^15^N values of tissues over time in some birds (Cherel *et al*., 2005), reptiles (McCue & Pollock, 2008) and humans (Mekota *et al*., 2006, 2009; Neuberger *et al*., 2013). The effects of starvation on *δ* ^13^C values are more complex but, in most cases, *δ* ^13^C values decrease with starvation (McCue & Pollock, 2008; Neuberger *et al*., 2013). Even without starvation, SIA has been used to detect changes in nutrition (Hobson *et al*., 1993; Castillo & Hatch, 2007; Williams *et al*., 2007; Deschner *et al*., 2012) and *δ* ^13^C and *δ* ^15^N values have been found to reflect changes in dietary composition over time (Petzke, Boeing & Metges, 2005; Huelsemann *et al*., 2009; Gillespie, 2013). If the health of an animal deteriorates at any point in its life, changes in stable isotope values of its tissues may occur, and SIA can potentially identify these changes. For illegally traded animals, *δ* ^13^C and *δ* ^15^N values of tissues from confiscated individuals can be investigated to determine whether there was a change from a natural diet to a more captive diet and results could be used to highlight the length of time that an animal had been held in captivity (although a good estimate of the tissue turnover rate would be required for this). Those being prosecuted for trafficking animals illegally may also be charged for violating animal welfare if aspects such as unsuitable diet or starvation are identified. In humans, some diseases/conditions (e.g. morning sickness, liver disease, diabetes, osteoporosis, cancer) affect stable isotope values, even without changes in diet (Fuller *et al*., 2005; Petzke *et al*., 2006; O'Grady *et al*., 2010; Reitsema, 2013; Tea *et al*., 2021). Animals in the wildlife trade are often affected by disease and have high mortality rates (Bezerra‐Santos *et al*., 2021; Cardoso *et al*., 2021). By investigating changes in isotope values, SIA can potentially be used to investigate the occurrence of disease in trafficked animals. There is currently very little information available on the health of animals within trafficking pathways and there is much scope for method development to help prevent the spread of disease.

## VI. LIMITATIONS AND RECOMMENDATIONS

While SIA has a number of benefits and shows great potential for use in investigating illegal wildlife trade (IWT) crimes and in wildlife forensics, there are some limitations that should be taken into consideration before wider application (Table 3). Below, we review these limitations and suggest where further research can help with developing SIA into a robust tool that can then be applied forensically.

**Table 3:** A summary of the limitations of stable isotope analysis (SIA) when applied to wildlife trade investigations and recommendations for overcoming these limitations.

1. Variation			
	Cause of variation	Examples of effects on wildlife trade investigations	Recommendations
**Within individuals**	– Differences within tissue types – Differences between tissue types – Temporal variations in diet – Movement between biomes – Speed of incorporation – Fractionation	**Captive/wild:** if a bird moults in captivity and new feathers are formed, these newer feathers may give a captive‐bred signal even though the bird was originally caught from the wild (Bearhop *et al*., 2003).	**General:** – further studies on tissue growth rates, isotope incorporation into tissues and fractionation – development of standardisation factors among different tissue types **Captive/wild:** – development of reference databases of both captive‐bred and wild‐caught individuals of species of interest, along with their food sources
**Within species/ between individuals**	– Age – Body size – Differences in diet/habitat – Different water sources	**Captive/wild:** can be complicated if species have large home ranges and diverse diets. Also complicated by differences in feed provided at different facilities for captive animals (Hopkins *et al*., 2022; Dufour *et al*., 2022). For example, some captive animals are fed a more ‘wild’ diet, e.g. wild rats/frogs (Natusch *et al*., 2017). **Geographic origin:** can be complicated in species with large geographic ranges and/or species that are ecological generalists and feed on a wide diversity of resources (Ziegler *et al*., 2016).	**Captive/wild:** – apply where species have small home ranges and/or specialised diets **Geographic origin:** – apply within certain perimeters to exclude unlikely areas of provenance
**Between different species**	– Differences in diet/habitat – Different water sources – Differences in geographic range	This variation can be used to advantage in wildlife trade investigations as it can result in significant differences between captive and wild individuals, individuals from different locations, and individuals from different species.	– can be used to investigate captive *versus* wild origin, provenance, and for species identification
**Environmental factors**	– Different biogeochemical processes – Climate – Altitude – Soil composition	As above	As above

2. Samples		
	Description of limitation	Recommendations
**Collection**	It is often necessary to sample opportunistically in wildlife trade investigations, which can create a bias.	– Development of reference databases
**Storage**	Sample quality is extremely important as compromised samples can lead to inaccurate and misleading results.	– Samples should be stored correctly and should not be stored in solvent as this can cause degradation that affects isotope values
**Preparation**	Different protocols and instrumentation can give rise to variation in results produced by different laboratories.	– Sample preparation methods should be kept consistent and should preferably be carried out in the same laboratory under the same conditions – If this is not possible, the same standards and references should be included
	Sample preparation is more complicated for some isotopes, e.g. hydrogen.	– Samples, reference materials and standards should all be equilibrated and analysed at the same time

### (1) Variation

The greatest limitation when using SIA for IWT investigations and/or for forensic casework is variation (Table 3). Natural variation in stable isotopes occurs at different scales (i.e. variation within individuals, among individuals, and among different species, as well as variation caused by differing environments). Each type of variation will affect investigative questions differently. Across all questions, studies dealing with variation within individuals require more focus on tissue growth/tissue turnover rates and testing the basics of isotope incorporation into tissues by, for example, carrying out controlled feeding experiments and measuring isotopic differences between different tissues. To date, these studies have largely focused on one or two species at a time [e.g. Japanese quail (*Cortunix japonica*) and American crow (*Corvus brachyrhynchos*) (Hobson & Clark, 1992); alpaca (*Lama pacos*) (Sponheimer *et al*., 2006)], but investigating these factors in a number of species in parallel would help to advance our knowledge. Further work should also be carried out to create standardisation factors to enable comparison between different tissue types, for example, different feather types, as done by Alexander *et al*. (2019).

### (2) Samples

When using SIA in IWT investigations and/or for forensic casework, care should be taken to ensure that all samples are collected, stored and prepared appropriately to prevent the unnecessary introduction of additional variation, enabling comparison of results across different processing runs and between different laboratories. In this field, it is often necessary to collect samples opportunistically, which can create a bias. It is important to build up reference databases of samples to fill gaps and reduce this bias (see Section (7)). Once collected, samples should be stored appropriately (and should ideally not be stored in solvent) to prevent degradation, which may affect stable isotope values. Sample preparation should be kept consistent and any treatments used should not alter the isotopic signal of the samples (Hobson *et al*., 2019b). Sample preparation for fixed tissues (e.g. feathers, hair, nails, claws, scales) that are tough and do not decompose easily is usually simpler and often involves cleaning with water alone. However, a solvent is recommended to dissolve and remove any surface oils, grease, or waxes that may be present and could affect stable isotope values (Wassenaar, 2019). Sample preparation for soft tissues (e.g. blood, muscle, liver) is often more complicated as the removal of lipids is usually essential due to the introduction of bias by lipids depleted in ^13^C and ^2^H (Wassenaar, 2019).

In addition, weighing of samples is extremely important and should be carried out on an analytical microbalance (with a readability of at least ±0.001 mg). Imprecise weighing can result in additional variance, particularly in *δ* ^2^H values (Wassenaar, 2019). Ideally, all preparation steps should be carried out in the same laboratory under the same conditions to enable comparison of results. If this is not possible, identical preparation and analysis steps should be followed and the same standards and references should be included to enable comparison of results among different laboratories (Dunn & Carter, 2018).

Sample preparation is more complicated for some isotopes, e.g. hydrogen. Hydrogen forms weak bonds with oxygen and nitrogen, therefore hydrogen that is not bound to carbon (i.e. hydrogen bonded to oxygen or nitrogen) in organic compounds may exchange isotopically with the hydrogen of ambient water vapour. This exchangeable hydrogen (DeNiro & Epstein 1981a; Schimmelmann, 1991; Wassenaar & Hobson, 2000) can affect *δ* ^2^H values and reduce the sensitivity when using hydrogen SIA to determine provenance (Wassenaar & Hobson, 2003; Meier‐Augenstein, Hobson & Wassenaar, 2013). Different laboratories in different locations can generate different results due to geographic and seasonal changes in the hydrogen isotopic content of ambient moisture and therefore it is difficult to compare results between laboratories (Wassenaar & Hobson, 2003). Reporting only values for non‐exchangeable hydrogen thus enables comparison of results between different laboratories. To do this, any exchange of hydrogen between ambient water vapour in the laboratory and exchangeable hydrogen in samples should be equalised over all samples and controls. The simplest method to address this is by using the comparative equilibrium approach, which has been used extensively for some animal tissues (e.g. hair keratin; Wassenaar & Hobson, 2003). Samples, reference materials and calibration standards are stored in the laboratory where analysis will take place and are allowed to equilibrate with ambient laboratory air moisture for >96 h prior to analysis. All samples, reference materials and standards should be in the same chemical form (i.e. are the same materials) and must be equilibrated and analysed at the same time so that the effect of external water is comparable between them (Wassenaar & Hobson, 2003; Meier‐Augenstein *et al*., 2011).

Addressing the abovementioned limitations is not only good scientific practice, but is also an important part of ensuring that scientific methods meet the criteria for appropriate wildlife forensic applicability for court (Moore *et al*., 2021). Together with the limitations and recommendations discussed above, there are additional important aspects that should be taken into account before undertaking an investigation using SIA or before using SIA to conduct forensic casework. These include the choice of isotope/s, the selection of appropriate tissues, standardisation and validation of SIA methods, construction of reference databases, and the inclusion of additional methods/techniques.

### (3) Choice of isotopes

The choice of isotope/s will depend on the aims of a study and also on access to specific instrumentation. In many studies, different isotopes will show variability and values have some overlap (i.e. due to overlapping habitats/geographic regions), therefore use of a single isotope is not always appropriate for discriminating between samples. By combining multiple isotopes it is possible to generate an isotopic fingerprint for the sample which can make these analyses more robust/precise (Ziegler *et al*., 2016).

### (4) Selection of appropriate tissues

#### (a) Variation in stable isotopes within tissue types

Variation can occur within a single tissue type at the time of sampling. For example, tissues with continuous growth (e.g. hair, scales, claws, feathers) will record information over a continuous period of time. Therefore, different sections of these tissues will represent different timeframes. Care should be taken to ensure that the sampling location is kept consistent when making comparisons across samples (Wassenaar, 2019). Very few studies have actually measured variation in stable isotopes within tissues for different plants/animals, although Ziegler *et al*. (2016) analysed samples along the length of two different elephant tusks and Pietersen *et al*. (2016) analysed isotopic data of samples taken along the length of two different pangolin scales. Considerable variation in stable isotope values was discovered between samples from the same tusk/scale, highlighting the importance of a consistent sampling location when comparing results across different samples. More studies investigating variation within different tissues for different species would be beneficial.

#### (b) Variation in stable isotopes between tissue types

Stable isotope values are often different between tissues due to various mechanisms including tissue turnover rate, metabolic routing, and isotopic fractionation. While variation between different tissue types can be a limitation of SIA, it can also be advantageous. By using a variety of tissues (or different sections of tissues with time‐dependent growth), detailed environmental/dietary histories can be constructed and geographic changes over time can be investigated. In captive/wild studies, it is possible to show the timeframe of release/capture by using different tissues. For example, in a study of mink (*Mustela vison*) in Denmark, a comparison of isotope values from several points along a continuously growing claw (relatively fast turnover rate) to those from teeth (slow turnover rate, representing a longer timeframe), allowed researchers to establish when a change of diet occurred, providing an estimate of when these mink had escaped from a farm (Hammershøj *et al*., 2005). In another study, comparison of isotope values of bone collagen and hair revealed the time of escape/release of wolves (*Canis lupus*) in the northeast USA (Kays & Feranec, 2011). Similarly, different plant tissues can provide temporal information. Plants retain information about their original location and changes can be investigated by comparing stable isotope values across different parts of the plant (Retief *et al*., 2014). It is, therefore, important to select tissues that represent the appropriate timeframe of interest and caution is needed when extrapolating SIA results between different tissue types within a single species.

Understanding isotopic incorporation into tissues, as well as isotopic fractionation, is also crucial for interpreting isotopic data (Wassenaar, 2019). Different tissues can reflect different dietary components due to differences in metabolism and routing. It is therefore important to understand the metabolism of different tissues when choosing the appropriate tissue for a study. For animals that moult inert tissues (e.g. fur, feathers) regularly, it is important to characterise the moulting period to clarify the timeframe that the tissues being investigated actually represent (Wassenaar, 2019).

### (5) Validation of SIA methods for use in wildlife forensics

Validation is a necessary step when transferring methods from research to forensics (Ogden *et al*., 2009). The process of designing and implementing validation studies for forensic genetic tests is well established (SWGDAM, 2016; Webster, Prigge & Frankham, 2024), but SIA has not been widely used in wildlife forensic science and there are currently no validated testing methods for its application therein. Further work will be required to ensure that methods are internationally recognised and standardised, enabling comparison of results among different laboratories (UNODC, 2014). To be used in this capacity, method validation is an essential step and should be carried out for SIA methods for all commonly traded species (Dunn *et al*., 2017). Method validation usually consists of experiments to test the repeatability, reproducibility, robustness, sensitivity, specificity, and resolution (SWGDAM, 2016; Webster *et al*., 2024). A key issue in genetic validation studies is reproducibility between different laboratories. For SIA, it may also be difficult to compare results among different laboratories due to the large number of factors that contribute to variation (e.g. differences between tissue types, temporal variation in diet, different sampling protocols). However, if identical procedures are followed and the appropriate standards and references are included, experiments could be comparable and reproducible. Part of the validation process should include trialling of methods using different materials that are commonly encountered in wildlife trade, including highly processed samples (e.g. tanned leather and cooked meat samples) and museum samples (which may have been chemically treated or preserved in formalin), to ensure that accurate results are obtained. During the validation process, all steps including sample collection, storage, preparation, mass spectrometry, and final data analysis should be examined carefully to avoid the introduction of unnecessary variation or, if unavoidable, to quantify that variation and develop protocols for correction (Ogden *et al*., 2009; Meier‐Augenstein, 2019; Brooks, Rugh & Werner, 2022). Once validated, methods should be applied with a secure chain of custody of evidence in a facility that meets the standards for quality control and quality assurance.

Laboratories with a forensic capacity should operate under strict quality management systems using validated testing methods to produce robust, reproducible and accurate results that can withstand questioning in courts of law (Ogden *et al*., 2009). International Standard Operating Procedures (SOPs) do exist for SIA and for the reporting of results, but these are not widely applied (Bond & Hobson, 2012; Roberts *et al*., 2018). SOPs are issued, regularly assessed and updated by the International Union of Pure and Applied Chemistry (IUPAC) and its Commission on Isotopic Abundances and Atomic Weights (Brand *et al*., 2014). Following these international SOPs ensures that results are both traceable to internationally accepted standards and are comparable between different laboratories (Meier‐Augustein, 2019). The forensic isotope ratio mass spectrometry (FIRMS) network (www.forensic-isotopes.org) was established in 2002 to develop the scope of stable isotope techniques in forensic applications. Members of this network conduct laboratory proficiency tests, provide guidance on suitable reference materials, help organise interlaboratory comparisons and evaluate the reliability of different analytical approaches (Cerling *et al*., 2016; Matos & Jackson, 2019). In 2011, members of the FIRMS network produced the “Good practice guide for isotope ratio mass spectrometry”, which provides guidance on instrumentation, quality assurance, troubleshooting and data management (Carter & Barwick, 2011). An updated version of this guide is now available (Dunn & Carter, 2018).

Introducing a protocol for quality control of SIA would help minimise variation in isotope values due to inconsistent methodology between laboratories. The World Anti‐Doping Agency (WADA) has established rules surrounding data used in antidoping proceedings and all samples can only be processed in WADA‐accredited laboratories. There is an External Quality Assessment Scheme (EQAS) where known blood/urine samples are distributed regularly to laboratories for analysis and the laboratory's accreditation is suspended if results are not accurate (WADA, 2021). A similar system could be introduced for wildlife forensic laboratories carrying out SIA to help ensure consistency across laboratories and over time. This could be administered in a way similar to that for the DNA Forensic Proficiency Testing Program run by the Society for Wildlife Forensic Science (https://www.wildlifeforensicscience.org/proficiency-testing/).

### (6) Standards

To enable interlaboratory comparison, it is important that all studies include appropriate standards to calibrate results so that they can be reported on the appropriate scale. If different laboratories use different calibration standards and/or procedures, additional variations will be introduced. It is, therefore, important that the same calibration procedures are adopted by all forensics laboratories. For calibration purposes, at least two certified standard reference materials of known composition must be included per element to normalise the raw data against international scales (Paul, Skrzypek & Fórizs, 2007; Roberts *et al*., 2018; Brooks *et al*., 2022). Internationally accepted standards of known isotopic composition are administered and supplied by internationally recognised organisations, e.g. the International Atomic Energy Agency (IAEA), the National Institute of Standards and Technology (NIST) and the United States Geological Survey (USGS) (Brand *et al*., 2014; Cerling *et al*., 2016). Many of the primary materials that were originally used as calibration standards no longer exist, however, secondary materials which have been calibrated to these primary materials can be used instead (Brand *et al*., 2014). It is important that these standards have internationally accepted values assigned to them which span the measurement range for the isotopes being analysed (Paul *et al*., 2007; Brooks *et al*., 2022). In addition to calibration standards, Quality Assurance (QA)/Quality Control (QC) standards should be measured alongside samples in every analysis to facilitate comparison of results between different runs within the same laboratory and also with other laboratories, both locally and internationally (Matos & Jackson, 2019). It is also recommended to include a standard for the species and tissue used in a study that can be run between samples to ensure that results remain consistent. In addition, it is important that investigators publishing SIA data calculate and present analytical uncertainty alongside their results (Szpak *et al*., 2017; Brooks *et al*., 2022). A simple method for calculating analytical uncertainty is provided by Szpak *et al*. (2017).

### (7) Reference databases

Wildlife and wildlife products that are traded through borders are often rare, elusive or endangered, therefore acquiring appropriate reference material from these animals or plants can be particularly challenging. Additionally, CITES regulations can complicate sample collection for reference use, especially if the species is not located domestically. To enhance reference material available for forensic and investigative studies, extensive databases should be developed for all species of interest. These databases should cover a broad geographic range, encompass various species and tissue types, and represent different time periods. Inclusion of environmental samples (e.g. soil and water) along with plant/animal samples from each geographic location would further enhance databases and would be useful for additional species, although laws and regulations regarding the import and export of soil or other material may prohibit this. In the absence of reference wildlife material, reference isoscapes assembled from environmental samples can be used to compare samples to predicted regions of origin (Bowen *et al*., 2005; West *et al*., 2010; Ehleringer *et al*., 2010). Although isoscapes have great potential for forensic application, there are some limitations. For example, there is considerable variability in the data available for different regions, both spatially and temporally. There is good geographic coverage over China, but collection of data there only began in the 1980s. Collection of data from America and Africa began much earlier, but had ceased in these regions by the time it began in China (www.waterisotopes.org; West *et al*., 2010). Some areas are underrepresented and isoscapes are less detailed. When using isoscapes, there are also underlying assumptions to be taken into account, e.g. not all animals incorporate hydrogen into their tissues from the water they drink, some obtain water (and therefore hydrogen) instead from their food or from the blood and/or urine of their prey (West *et al*., 2010; Chesson *et al*., 2018; Meier‐Augenstein, 2019). Therefore, calibration data from reference samples of known origin may be required to interpolate data from isoscapes to species of interest. These samples should be used to verify the presence of a strong relationship between the stable isotope values of the animal tissue and environmental samples and that the interpolations are accurate (Hobson, 1999).

Reference databases can be assembled by researchers from different fields, working on different taxa and/or those conducting environmental studies. Organising such work is energy‐, resource‐, and time‐consuming, however, leveraging personnel across applicable fields to conduct sampling should not be impossible, and has been done previously for other methods (e.g. in genetics, the ForCyt database contains mitochondrial DNA sequences of species commonly encountered in forensic investigations; Ahlers *et al*., 2017). Construction of isotopic reference databases could be supported by the establishment of international platforms for data collection that could be monitored and curated through in‐country groups of researchers or under the umbrella of a larger international network to ensure standardisation and reliability. For example, IsoBank is a fairly new repository for isotopic data involving collaboration between groups from different universities in the USA and funded by the National Science Foundation (www.isobank.org; Shipley *et al*., 2024). IsoBank has recently started accepting data sets, but the number of data sets available is still limited and, as with any repository built upon submissions from the public, one should be aware of potential data error and all metadata should be thoroughly evaluated before data sets are used (www.isobank.org; Shipley *et al*., 2024). It is important to consider the metadata and ensure that methods are standardised within databases to enable sharing and comparison of data between different laboratories, to develop international capacity for researchers without access to samples, and to allow for method validation – an important part of ensuring scientific tools are suitable for forensic application.

### (8) A toolkit of multiple forensic techniques

Through the integration of multiple approaches, a range of questions can be answered and more precise data can be generated. As an example, when investigating the breeding origin of migrant birds, a combination of SIA, habitat suitability models and genetic data enabled misassignments (based on genetic data alone) to be resolved and assignments to be made with increased accuracy (Ruegg *et al*., 2017). It has been demonstrated that results obtained using a combination of methods were more conclusive than those from individual methods alone (Ruegg *et al*., 2017). The inclusion of other methods (e.g. morphology, genetics, and/or elemental methods) alongside SIA, can increase the accuracy of the conclusions, generating robust data appropriate for forensic purposes. Many studies mentioned herein used additional methods in conjunction with SIA to obtain more robust/precise findings. For example, combining elemental markers with SIA enabled discrimination between diets and provenance with up to 100% accuracy in pythons in Vietnam and Indonesia (Natusch *et al*., 2017). In addition, CSIA allowed discrimination in cases where SIA results alone were inconclusive when investigating captive *versus* wild cockatoos in Hong Kong (Andersson *et al*., 2021a). When SIA is unable to resolve populations across large geographic ranges, the inclusion of other methods can increase the resolution and improve assignment accuracy.

## VII. CONCLUSIONS

(1) SIA is a dynamic tool for investigating wildlife crimes that can be further developed for broader application in wildlife forensics.
(2) Although SIA is unlikely to replace existing methods such as genetic or morphological methods for species identification, it has substantial potential to complement these techniques in overcoming certain constraints (e.g. low DNA quality) and could provide important additional information.
(3) Current research has demonstrated the application of SIA in investigating wildlife trade, addressing questions related to species identification, determination of provenance, and captive *versus* wild source differentiation.
(4) Additional potential applications of SIA to IWT research include, but are not limited to, individual tracing techniques and investigations into health and animal welfare along trafficking pathways. We encourage more research into novel applications of SIA to wildlife trade questions.
(5) Recommendations to ensure the robustness of SIA results in courts as legal evidence include (*i*) the development of validated SIA methods to produce data calibrated to internationally recognised standards and reference materials, and (*ii*) collaborations between research groups and forensic scientists to develop SIA further for forensic use.

## Supporting information


**Table S1.** Values (in US$) and references used for price per sample estimations for the different methods included in Table 1.
**Table S2**. Summary of previous studies where stable isotope analysis has been used successfully for species identification/differentiation.
**Table S3**. Selected previous studies where stable isotope analysis has been used successfully to differentiate between habitats/investigate geographic provenance.
**Table S4**. Summary of a selection of studies where stable isotope analysis has been used successfully to differentiate between captive/cultivated and wild individuals.
**Table S5**. Examples of studies examining the effect of different environmental/nutritional stressors or health conditions on the stable isotope values of human and animal tissues.
