# Supplementary material for: Wildlife trade investigations benefit from multivariate stable isotope analyses
Source: Biol Rev Camb Philos Soc. 2024 Dec 27;100(3):1083–104. doi: 10.1111/brv.13175 (PMC12120388; doi:10.1111/brv.13175)
Supplement: Supplementary file 1 — Table S1. Values (in US$) and references used for price per sample estimations for the different methods included in Table 1. Table S2. Summary of previous studies where stable isotope analysis has been used successfully for species identification/differentiation. Table S3. Selected previous studies where stable isotope analysis has been used successfully to differentiate between habitats/investigate geographic provenance. Table S4. Summary of a selection of studies where stable isotope analysis has been used successfully to differentiate between captive/cultivated and wild individuals. Table S5. Examples of studies examining the effect of different environmental/nutritional stressors or health conditions on the stable isotope values of human and animal tissues. [file BRV-100-1083-s001.docx]

**Table S1.** Values (in US$) and references used for price per sample estimations for the different methods included in Table 1. The ranges given in Table 1 were compiled from the lowest possible cost and highest possible cost identified.

| **Stable isotope analysis^a^** | **Genetics^b^** | **Morphology^c^** | **X-ray fluorescence** | **Reference** |
| --- | --- | --- | --- | --- |
| $15–30 | – | – | – | https://www.biosch.hku.hk/si_lab/* |
| – | $8 (per sample for bi-directional Sanger sequencing with a single primer set for barcoding) | – | – | https://cpos.hku.hk/portfolio-item/sanger-dna-sequencing/#service_charges * |
| $7–200 | – | – | – | Wassenaar (2019) |
| $100–400 | $100–300 | <$100 | – | Dormontt *et al*. (2015) |
| – | – | – | $130 | https://www.crb-gmbh.com/en/pricelists/pricelist-xrf-analysis |

*Services used by the author.

^a^Can vary greatly depending on the number of stable isotopes analysed.

^b^Can range from simple barcoding for species identification to population genetics/phylogeography for provenance determination.

^c^May be necessary to prepare slides/use microscopy.

**Table S2.** Summary of previous studies where stable isotope analysis has been used successfully for species identification/differentiation.

| **Species** | **Isotopes used** | **Tissues used** | **Reference** |
| --- | --- | --- | --- |
| Atlantic cod *Gadus morhua*  Saithe *Pollachius virens* | *δ*^13^C and *δ*^15^N | Bone, skin, muscle | Oliviera *et al*. (2011) |
| Balearic shearwater *Puffinus mauretanicus*  Yelkouan shearwater *Puffinus yelkouan* | *δ*^13^C and *δ*^15^N | Feathers | Militão *et al*. (2014) |
| Scallops *Patinopecten yessoensis*, *Chlamys farreri* and *Argopecten irradians* | *δ*^13^C and *δ*^15^N | Adductor muscle | Zhang *et al*. (2019) |
| Cedar trees *Cedrela* *odorata* and *Cedrela fissilis* | *δ*^13^C and *δ*^18^O | Wood | Paredes-Villanueva *et al*. (2022) |

**Table S3.** Selected previous studies where stable isotope analysis has been used successfully to differentiate between habitats/investigate geographic provenance.

| **Species** | **Isotopes used** | **Tissues used** | **Reference** |
| --- | --- | --- | --- |
| 43 East African mammal species | *δ*^13^C and *δ*^15^N | Bone collagen | Ambrose & DeNiro (1986) |
| African elephant *Loxodonta africana* | *δ*^13^C | Ivory | van der Merwe *et al*. (1988) |
| African elephant *Loxodonta africana* | *δ*^13^C, *δ*^15^N, ^87^Sr/^86^Sr, and several Pb ratios | Bone, ivory | Vogel *et al*. (1990) |
| 25 avian species | *δ*^2^H and *δ*^18^O | Feathers | Hobson *et al*. (2004) |
| African elephant *Loxodonta africana*  Hippopotamus *Hippopotamus amphibius* | *δ*^13^C and *δ*^18^O | Ivory, bioapatite | Cerling *et al*. (2007) |
| 12 tree species | *δ*^2^H | Wood (lignin methoxyl groups) | Keppler *et al.* (2007) |
| Rubroshorea wood 10 *Shorea* spp. | *δ*^13^C, *δ*^15^N and *δ*^18^O | Wood | Kagawa *et al*. (2007) |
| Goldfinches *Carduelis carduelis britannica* and *Carduelis carduelis* [*frigoris*] *major* | *δ*^2^H | Feathers | Kelly *et al*. (2008) |
| Larch wood *Larix* sp. | *δ*^13^C and *δ*^18^O | Wood | Horacek *et al*. (2009) |
| Pinyon pines *Pinus edulis* and *Pinus monophylla* | *δ*^13^C | Wood | Kagawa & Leavitt (2010) |
| Ovenbird *Seiurus aurocapilla* | *δ*^2^H | Feathers | Haché *et al*. (2012) |
| Elephants *Loxodonta africana* and *Elephas maximus* | *δ*^13^C, *δ*^15^N, *δ*^34^S, *δ*^18^O, and *δ*^2^H | Ivory | Ziegler *et al.* (2016) |
| Burmese python *Python bivittatus*  Reticulated python *Python reticulatus* | *δ*^13^C, *δ*^15^N and *δ*^2^H | Skin | Natusch *et al*. (2017) |
| Frogs *Hoplobatrachus rugulosus*, *Fejervarya cancrivora* and *Limnonectes macrodon* | *δ*^13^C, *δ*^15^N and *δ*^18^O | Muscle tissue, bone | Dittrich *et al*. (2017) |
| African grey parrot *Psittacus erithacus* | *δ*^13^C, *δ*^15^N and *δ*^2^H | Feathers | Alexander *et al*. (2019) |
| Ortolan bunting *Emberiza hortulana* | *δ*^2^H | Feathers | Jiguet *et al*. (2019) |
| African elephant *Loxodonta africana* | *δ*^13^C, *δ*^15^N and *δ*^34^S | Ivory | Hale *et al*. (2021) |
| Cedar trees *Cedrela* *odorata* and *Cedrela fissilis* | *δ*^13^C and *δ*^18^O | Wood | Paredes-Villanueva *et al*. (2022) |
| Azobé *Lophira alata*  Tali *Erythrophleum ivorense* and *Erythrophleum suaveolens* | *δ*^13^C, *δ*^34^S, *δ*^18^O, and *δ*^2^H | Wood | Boeschoten *et al*. (2023) |
| Palawan forest turtle *Siebenrockiella leytensis*  Philippine cockatoo *Cacatua haematuropygia*  Philippine pangolin *Manis culionensisis* | *δ*^13^C and *δ*^15^N | Scutes, feathers, claws, scales | Brandis *et al*. (2023) |
| Cheetah *Acinonyx jubatus* | *δ*^13^C, *δ*^15^N, *δ*^18^O, and *δ*^2^H | Hair | Koehler *et al*. (2023) |

**Table S4.** Summary of a selection of studies where stable isotope analysis has been used successfully to differentiate between captive/cultivated and wild individuals. Additional methodologies/tools used are also included.

| **Species** | **Isotopes used** | **Tissues Used** | **Additional methodology/**  **tools used** | **Reference** |
| --- | --- | --- | --- | --- |
| Atlantic salmon *Salmo salar* | *δ*^13^C and *δ*^15^N | Adipose tissue, muscle tissue | – | Dempson & Power (2004) |
| American mink *Mustela vison* | *δ*^13^C | Teeth, claws | Microsatellites | Hammershøj *et al*. (2005) |
| Coyote *Canis latrans*  Wolf *Canis lupus* | *δ*^13^C and *δ*^15^N | Bone collagen, hair | – | Kays & Feranec (2011) |
| Lebombo cycad *Encephalartos lebomboensis*  Alexandria cycad *Encephalartos arenarius* | *δ*^15^N, *δ*^34^S, *δ*^18^O and ^87^Sr/^86^Sr | Petiole  Upper, middle and lower leaf base  Upper core  Middle core inner and middle core outer  Lower core inner, lower core middle and lower core outer | ^14^C analysis (for dating) | Retief *et al*. (2014) |
| Crocodile lizard *Shinisaurus crocodilurus*  Monitor lizards *Varanus acanthurus*, *Varanus macraei*, *Varanus melinus* and  *Varanus salvator* | *δ*^13^C and *δ*^15^N | Skin, faeces | Feeding experiments with a ^15^N-enriched marker | Crook *et al*. (2016) |
| Crocodile lizard *Shinisaurus crocodilurus* | *δ*^13^C and *δ*^15^N | Tail clippings | – | van Schingen-Khan *et al*. (2016) |
| Burmese python  *Python bivittatus*  Reticulated python  *Python reticulatus* | *δ*^13^C, *δ*^15^N and *δ*^2^H | Skin | Elemental markers | Natusch *et al*. (2017) |
| Frogs *Hoplobatrachus rugulosus*,  *Fejervarya cancrivora*, and *Limnonectes macrodon* | *δ*^13^C, *δ*^15^N and *δ*^18^O | Muscle tissue, bone | DNA barcoding (for species confirmation) | Dittrich *et al*. (2017) |
| Northern bobwhite  *Colinus virginianus* | *δ*^13^C, *δ*^15^N, *δ*^34^S, and *δ*^2^H (one preliminary sample only) | Feathers | – | Castelli & Reed (2017) |
| Short-beaked echidna  *Tachyglossus aculeatus* | *δ*^13^C and *δ*^15^N | Quills | High-resolution X-ray fluorescence (XRF) | Brandis *et al*. (2018) |
| African grey parrot  *Psittacus erithacus* | *δ*^13^C, *δ*^15^N and *δ*^2^H | Feathers | – | Alexander *et al*. (2019) |
| Ortolan bunting  *Emberiza* *hortulana* | *δ*^2^H | Feathers | – | Jiguet *et al*. (2019) |
| Red-eared slider turtle *Trachemys scripta elegans* | *δ*^13^C and *δ*^15^N | Scutes | – | Hill *et al*. (2020) |
| Yellow-crested cockatoo *Cacatua sulphurea* | *δ*^13^C and *δ*^15^N | Feathers | Compound specific isotope analysis (CSIA) | Andersson *et al*. (2021*a*) |
| Tokay gecko *Gekko gecko gecko* and  *Gekko gecko reevesii* | *δ*^13^C and *δ*^15^N | Tail clippings | Genetics (barcoding and phylogenetic analysis) | Dufour *et al*. (2022) |
| Wood turtle  *Glyptemys insculpta* | *δ*^13^C and *δ*^15^N | Claw tips | – | Hopkins *et al*. (2022) |
| Palawan forest turtle  *Siebenrockiella leytensis*  Philippine cockatoo  *Cacatua haematuropygia* | *δ*^13^C and *δ*^15^N | Scutes, feathers | Portable X-ray fluorescence (pXRF) | Brandis *et al*. (2023) |

**Table S5.** Examples of studies examining the effects of different environmental/nutritional stressors or health conditions on the stable isotope values of human and animal tissues.

| **Species** | **Treatment/**  **illness** | **Tissues used** | **Isotope used** | **Effect on isotope values** | **Reference** |
| --- | --- | --- | --- | --- | --- |
| Japanese quail  *Coturnix japonica*  Ross’ geese *Chen rossii* | Fasting/  nutritional restriction | Muscle, liver, blood, feathers, bone collagen | *δ*^15^N | Enrichment of *δ*^15^N, *δ*^13^C unchanged | Hobson *et al*. (1993) |
| Bats  *Glossophaga soricine*; *Leptonycteris curasoae* | Nitrogen-poor  diet with a high *δ*^15^N and *δ*^13^C (low protein diet) | Blood, wing membrane | *δ*^13^C and *δ*^15^N | Enrichment of *δ*^15^N and *δ*^13^C for both blood and wing membrane | Voigt & Matt (2004) |
| King penguin *Aptenodytes patagonicus* | Fasting | Blood, feathers | *δ*^13^C and *δ*^15^N | Higher *δ*^15^N values, decrease in *δ*^13^C values in plasma | Cherel *et al*. (2005) |
| Human  *Homo sapiens* | Morning sickness (nutritional stress) | Hair | *δ*^15^N | Elevated *δ*^15^N, no change in *δ*^13^C | Fuller *et* *al*. (2005) |
| Human  *Homo sapiens* | Anorexia nervosa (starvation) | Hair | *δ*^13^C and *δ*^15^N | Increase in *δ*^15^N values, decrease in *δ*^13^C values during starvation;  decrease in *δ*^15^N values, increase in *δ*^13^C values during recovery | Mekota *et al*. (2006, 2009) |
| Human  *Homo sapiens* | Liver disease | Hair | *δ*^15^N | Lower *δ*^15^N values | Petzke *et al*. (2006) |
| Side-blotched lizard  *Uta stansburiana*  Green anole  *Anolis carolinensis* | Fasting/  nutritional restriction | Uric acid,  tail tissue | *δ*^15^N | Higher *δ*^15^N values in uric acid; no difference in tail tissue | Castillo & Hatch (2007) |
| Tufted puffin  *Fratercula cirrhata* | Fasting/  nutritional restriction | Blood | *δ*^13^C and *δ*^15^N | Lower *δ*^15^N and *δ*^13^C values | Williams *et al*. (2007) |
| Song sparrow  *Melospiza melodia* | Nutritional stress | Blood, liver,  muscle,  feathers | *δ*^13^C and *δ*^15^N | No significant effect | Kempster *et al*. (2007) |
| Gaboon viper *Bitis gabonica*;  Ball python *Python regius;*  Ratsnake *Elaphe obsolete*;  Boa constrictor *Boa constrictor*;  Western diamondback rattlesnake *Crotalus atrox*;  Savannah monitor lizard *Varanus exanthematicus* | Starvation | Faeces, carcass, scales, claws | *δ*^13^C and *δ*^15^N | Little change in values in carcasses; *δ*^15^N values in excreta were higher and *δ*^13^C values in excreta were lower; values in scales and claws less indicative of starvation time | McCue & Pollock (2008) |
| Human *Homo sapiens* | Change in diet  (C3→C4 plant, terrestrial animal products →marine animal products) | Hair | *δ*^13^C and *δ*^15^N` | Increase in *δ*^15^N and *δ*^13^C values | Huelsemann *et al*. (2009) |
| Mouse *Mus musculus* | Diabetes | Body water | *δ*^18^O and *δ*^2^H | *δ*^18^O and *δ*^2^H values of body water from diabetic mice were closer to those of drinking water when compared to control mice | O’Grady *et al.* (2010) |
| Bonobo *Pan paniscus* | Fasting/  nutritional restriction | Urine | *δ*^13^C and *δ*^15^N | Increase in *δ*^13^C and *δ*^15^N values | Deschner *et al*. (2012) |
| Human *Homo sapiens* | Applied to forensic cases of severe starvation prior to death in neglect and abuse cases in children and the elderly | Hair | *δ*^13^C and *δ*^15^N | Increase in *δ*^15^N values, decrease in *δ*^13^C values | Neuberger *et al*. (2013) |
